# Limited airway resection and reconstruction for paediatric tracheobronchial inflammatory myofibroblastic tumour

**DOI:** 10.1093/icvts/ivac117

**Published:** 2022-05-06

**Authors:** Junguo Dong, Diego Gonzalez-Rivas, Pengcheng Lv, Zhexin Wang, Jiaxi He, Feng Yao, Shuben Li

**Affiliations:** 1 Department of Thoracic Surgery and Oncology, the First Affiliated Hospital of Guangzhou Medical University, State Key Laboratory of Respiratory Disease, National Clinical Research Center for Respiratory Disease, Guangzhou Institute of Respiratory Health, Guangzhou, China; 2 Department of Thoracic Surgery, Coruña University Hospital, Coruña, Spain; 3 Department of Thoracic Surgery, Shanghai Chest Hospital, Shanghai Jiao Tong University, Shanghai, China

**Keywords:** Tracheobronchial inflammatory myofibroblastic tumour, Paediatric, The long-term prognosis

## Abstract

**OBJECTIVES:**

The paediatric tracheobronchial inflammatory myofibroblastic tumour (IMT) is a rare disease. Whether limited surgical resection is a feasible surgical approach for these patients remains controversial. The objectives of this study were to report the long-term prognosis after limited surgical resections on paediatric tracheobronchial IMT and provide a surgical management strategy for this rare disease.

**METHODS:**

Paediatric tracheobronchial IMT patients who underwent limited surgical resection from 2012 to 2020 were enrolled in this study. The clinical characteristics, course of treatment and long-term outcomes of all participants were collated. We presented the accumulated data and analysed the feasibility of limited surgical resection on the paediatric tracheobronchial IMT.

**RESULTS:**

A total of 9 children with tracheobronchial IMTs were enrolled in our study. Cough and shortness of breath were the most common symptoms. All 9 participants underwent surgical treatment, including 2 tracheal reconstructions, 4 carinal reconstructions and 3 bronchial sleeve resections. Among the participants, 6/9 (66%) were positive for the anaplastic lymphoma receptor tyrosine kinase gene in terms of immunohistochemistry. None of the participants died of short-term complications. The follow-up period was 5.4 (range, 1.1–9.3) years, during which all participants remained well.

**CONCLUSIONS:**

Limited surgical resection is preferred for paediatrics with tracheobronchial IMTs. Meanwhile, patients with complete resection have an excellent long-term prognosis.

**Figure:**
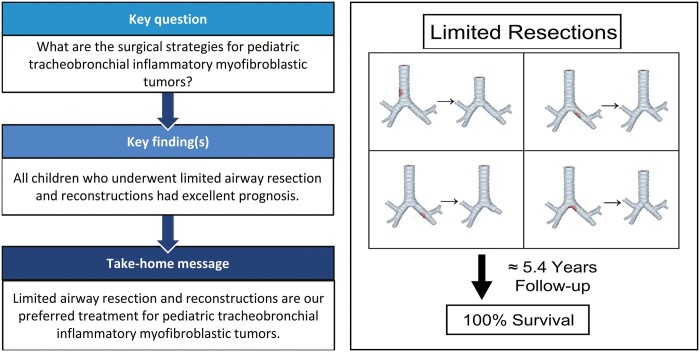

## INTRODUCTION

Inflammatory myofibroblastic tumour (IMT) is an uncommon disease. It is typically considered a low-grade malignant, locally invasive malignant tumour with a low recurrence and metastasis rate [1]. Paediatric tracheobronchial IMT is exceedingly rare, and experience in treating paediatric tracheobronchial IMT is lacking. Complete lung function is significant for children's growth and quality of life. Surgical resection for IMT includes even pneumonectomy to prevent a recurrence. However, these surgical approaches entail an excessive loss of lung parenchyma and may lead to severe complications. Limited surgical resection is a set of surgical approaches, which remove the lesion and preserve more pulmonary parenchyma, including tracheal reconstruction, carinal reconstruction, bronchial sleeve resection and sleeve lobectomy. At present, studies about paediatric tracheobronchial IMT with limited surgical resections have mostly been case reports [2, 3]. We aimed to elucidate whether limited surgical resection can also prevent recurrence and achieve long-term survival. This study collected 9 patients with tracheobronchial IMT with limited surgical resections. The objectives of this study were to present the long-term prognosis after limited surgical resections on paediatric tracheobronchial IMT and provide a surgical management strategy for this rare disease.

## PATIENTS AND METHODS

**Figure 1:**
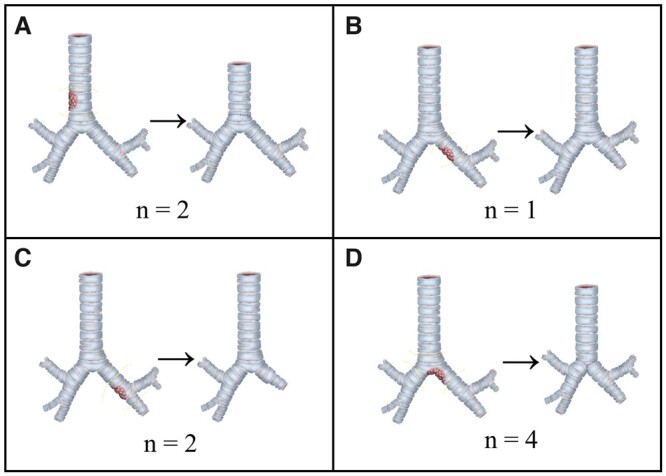
Different tumour locations and their corresponding limited surgical resection methods. (**A**) Tracheal reconstruction. (**B**) Bronchial sleeve resection. (**C**) Sleeve lobectomy. (**D**) Carinal reconstruction.

**Figure 2:**
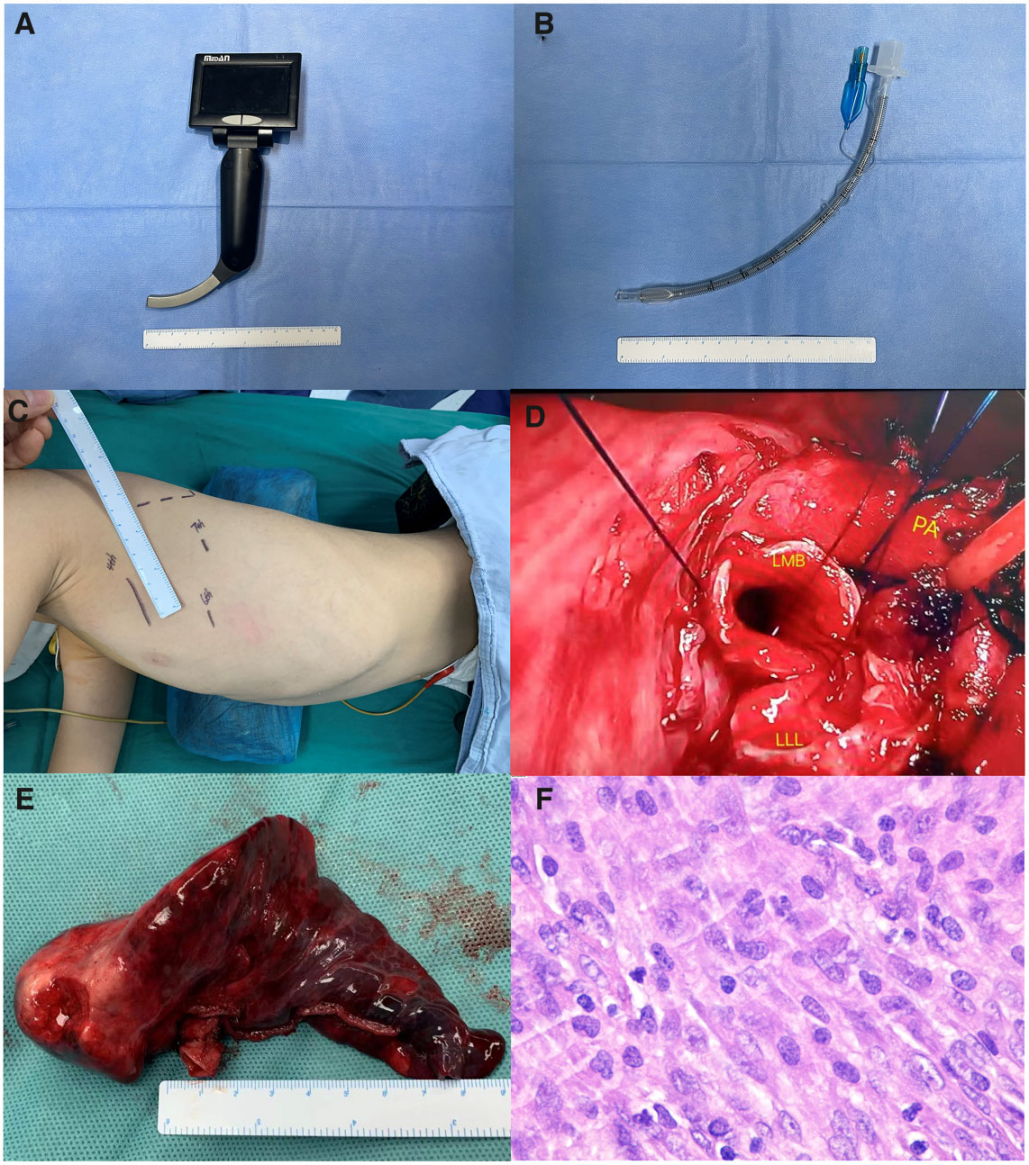
Surgery pictures. (**A**) Visual laryngoscope for paediatric. (**B**) Tracheal intubation cannula for paediatric. (**C**) Surgical approaches. (**D**) Sleeve lobectomy. (**E**) Lesion specimen. (**F**) Pathological picture. LLL: left lower lobe; LMB: left main bronchi; PA: pulmonary artery.

This retrospective series report recruited consecutive 9 paediatric tracheobronchial IMT patients with limited surgical resection from the First Affiliated Hospital of Guangzhou Medical University and Shanghai Chest Hospital in China between 2012 and 2020. The clinical characteristics and preliminary diagnosis of all participants were recorded. Participants underwent preoperative pulmonary computed tomography (CT) scan. The majority underwent preoperative bronchoscopic biopsy. None of the participants received preoperative neoadjuvant therapy. Preparation before intubation includes selecting a double lumen tube with appropriate model and direction, checking the inflation/deflation of bronchial and tracheal inflation cuff, preparing a special Y-connector and connecting double lumen and ventilation system. Intubation requires a skilled direct visual laryngoscope (lifting the epiglottis) to expose the throat fully. The left double-lumen tube also needs to rotate 90° clockwise to place the front end at an angle in the correct position. Once the double-lumen tube passes through the throat, pull out the tube core and push the double-lumen tube to the lower part of the trachea to make the bronchial tube enter the correct bronchus. One patient with a tumour located in the middle trachea and one with a tumour located in the lower trachea received VATS tracheal reconstruction via the right approach and the sternal approach, respectively (Fig. 1A). Three patients with tumours located at the distal left main bronchus underwent sleeve lobectomy or bronchial sleeve resection (Fig. 1B and C). Four patients with tumours involving the carina and proximal bronchus underwent carina reconstructions (Fig. 1D). The resection margin was 5 mm away from the lesion. The result of the frozen section determined extended resection. They used the suspension wire to test the anastomotic tension and select the angle and method of anastomosis. The anastomosis was continuously sutured by polydioxanone plus 4–0 and then intermittently sutured by Vicryl 4–0 for reinforcement. Postoperatively, the whole tumour specimen was histologically evaluated, and the final diagnosis was determined by the paraffin section. Pathologists provide all reports regarding histological diagnoses (Fig. 2). Participants were followed up by the outpatients at the first 6 months and annually after surgery. The last follow-up was performed by telephone.

### Carinal reconstruction technique

There were 4 patients with tumours involving the carinal and proximal bronchus underwent carinal reconstructions. Tissue around the trachea was fully released, and the incision was made 5 mm from the upper and lower edges of the lesion. Right main bronchus were resected 5 mm from the orifice. The lower part of the trachea and the left main bronchus were suspended respectively, and the artificial carina was formed by continuous suture. The excess tissue of the artificial carina was trimmed. After that, the right main bronchi and the gap were continuously sutured. The anastomoses were sutured interruptedly for reinforcement.

### Ethics statement

This study was permitted by the Ethics Committee of the First Affiliated Hospital of Guangzhou Medical University (2020K43). Since this was a retrospective study and all participants were anonymous, individual informed consent was waived.

### Statistical analysis

Data were analysed using the SPSS software version 26.0 (IBM Corp., Armonk, NY, USA). Continuous variables were expressed by median and interquartile range (IQR), since it was not in a normal distribution. Categorical variables were expressed by absolute frequencies.

## RESULTS

A total of 9 patients with tracheobronchial IMTs were enrolled in our study. There was no gender difference in our cases, with 4 male patients and 5 female patients. The median age was 12 (IQR, 9–13) years old. The median height and weight were 144 (IQR, 128–156) cm and 39 (IQR, 25–44) kg, respectively. The symptoms were varied as determined by the specific location of the lesion. Among the 9 participants, the most common symptoms were cough (6/9, 66.7%) and shortness of breath (6/9, 66.7%). Other local symptoms included dyspnoea (5/9, 55.6%), chest distress (5/9, 55.6%), chest pain (3/9, 33.3%) and haemoptysis (1/9, 11.1%). Besides the local specific symptoms, some common systemic symptoms were observed, including fever (2/9, 22.2%). The median time of symptomatic onset to diagnosis was 30 (IQR, 21–45) days.

In terms of the preoperative CT scans, patients presented as atelectasis (4/9, 44.4%), lung infection (5/9, 55.6%), pleural effusion (4/9, 44.4%) and airway obstruction (5/9, 55.6%). Of the 9 participants, 8 (8/9, 88.9%) underwent preoperative bronchoscopy and 5 of 8 (62.5%) were initially diagnosed with IMT (Table 1).

**Table 1:** Patient characteristics

Case No.	Age (years), sex	Height (cm), weight (kg)	Symptoms	Imaging characteristics	Tumour location, size (cm)
1	7, M	128, 21.1	CD, CP, SB	LI, PE, atelectasis	Distal LMB, 3
2	15, M	160, 39	C, D, P, CP CD, SB, fever	LI, PE, atelectasis	Carina + proximal LMB, 3
3	10, M	140, 26	C, H	No	Carina + proximal RMB, 2
4	9, F	127, 24	C, P, CD, CP, SB	LI, PE, atelectasis	Distal LMB, 2.5
5	16, F	144, 42	D, P, SB	No	Trachea, mid, 1.9
6	6, M	125, 25	C, D, P, CD, SB, fever	LI	Trachea, low, 1
7	12, F	153, 44	C, D	No	Carina + proximal LMB, 2.5
8	13, F	156, 45	C, P	No	Carina + proximal LMB, 1.5
9	13, F	160, 65	D, CD, SB	LI, PE, atelectasis	Distal LMB, 3.4
Median	12	144, 39			2.5

C: cough; CD: chest distress; CP: chest pain; D: dyspnoea; F: female; H: haemoptysis; SB: shortness of breath; LI: lung infection; LMB: left main bronchi; M: male; mid: middle; no.: number; P: palpitation; PE: pleural effusion; RMB: right main bronchi.

**Table 2:** Surgery and follow-up information

Case no.	Approach	Surgical technique	Blood loss (ml)	Hospital stay (days)	Margin	Recurrence	Survival (years)
1	Left VATS	LUL sleeve lobectomy	50	10	R0	No	1.1, alive
2	Left thoracotomy	Carinal reconstruction	150	9	R0	No	4.5, alive
3	Left VATS	Carinal reconstruction	30	13	R0	No	4.5, alive
4	Left VATS	LMB sleeve resection	30	16	R0	No	5.4, alive
5	Sternotomy VATS	Tracheal reconstruction	5	14	R0	No	8.6, alive
6	Right VATS	Tracheal reconstruction	50	13	R0	No	9.3, alive
7	Right VATS	Carinal reconstruction	700	44	R0	No	5.4, alive
8	Right Thoracotomy	Carinal reconstruction	100	11	R1	Yes	6.5, alive
9	Left Thoracotomy	LUL sleeve lobectomy	200	5	R0	No	3.3, alive
Median			50	14			5.4

LMB: left main bronchus; LUL: left upper lobe; no.: number; R0: microscopically and macroscopically margin-negative; R1: macroscopically margin-negative, but microscopically margin-positive; RMB: right main bronchus; VATS: video-assisted thoracoscopic.

None of the participants received neoadjuvant therapy before surgery. All 9 participants underwent surgical treatment. Two participants with tracheal tumour received tracheal reconstructions. Four participants with tumours involving the carina and proximal main bronchus underwent carinal reconstructions. Two participants with main bronchial tumour extending to the opening of lobar bronchus received sleeve lobectomies. One participant with main bronchial tumour not involving with the opening of lobar bronchus underwent bronchial sleeve resection. The median tumour diameter was 2.5 (IQR, 1.9–3) cm. Tumour-free surgical margin was recorded in 8 participants.

The CT scans and bronchoscopy examinations of each participant were evaluated after surgery. Postoperative complications (5/9, 55.6%) included postoperative atelectasis (4/9, 44.4%), empyema (1/9, 11.1%) and lung infection (3/9, 33.3%). After surgery, the median hospital stay was 13 (IQR, 10–14) days. None of the participants died of short-term complications.

The follow-up data were available for 5.4 (IQR, 4.5–6.5) years, during which time all participants remained well. None of the participants received adjuvant therapy after surgery. All participants underwent postoperative CT scans and bronchoscopy examinations on time. A case suspected of a local recurrence after the operation and underwent bronchoscopic tumour resection and airway dilation. This patient did not recur again and was still alive at the last follow-up (Table 2).

The pathological specimens were checked by 2 experienced pathologists. All participants were confirmed with tracheobronchial IMTs. The immunohistochemistry (IHC) of the postoperative pathological specimens were analysed, and the results were as follows: 6/9 (66%) was positive for the anaplastic lymphoma receptor tyrosine kinase gene (ALK), 9/9 (100%) were positive for vimentin and 7/9 (77.8%) were positive for alpha-smooth muscle actin. Seven patients had S-100 positive (7/9, 77.8%), while only 2 and 3 patients had positive desmin (2/9, 22.2%) and CD34 (3/8, 27.5%). None of their samples expressed CK in this series (Table 3).

**Table 3:** Characteristics of immunohistochemistry

Case no.	Vimentin	ALK	SMA	Desmin	S-100	CD34	CK
1	+	+	+	+	+	+	–
2	+	+	−	−	−	+	−
3	+	+	+	−	+	−	−
4	+	+	+	−	+	N/A	−
5	+	−	+	+	+	−	−
6	+	−	+	−	+	−	−
7	+	−	+	−	+	+	−
8	+	+	+	−	+	−	−
9	+	+	−	−	−	−	−
Positive rate (%)	100	66	77.8	22.2	77.8	37.5	0

N/A: not available; no.: number; SMA: smooth muscle actin.

## DISCUSSION

The first report of IMT was by Brunn in 1939, and it was named in 1954 in accordance with its propensity to clinically and radiologically mimic a malignant process [4]. It is a low-grade malignant tumour that originated from soft tissue, which predates paediatrics, and mainly grows in the lung. Paediatric tracheobronchial IMT is extremely rare. Available articles about the paediatric tracheobronchial IMT cohort are limited, and most are case reports. This study is a retrospective observational analysis of paediatric tracheobronchial IMT and provides a detailed overview of patients’ clinical characteristics, auxiliary examinations, treatment history and long-term prognosis.

Paediatric tracheobronchial IMT presents with non-specific symptoms. The initial symptoms of patients in this study were mainly cough, dyspnoea, shortness of breath, chest pain and fever. The period of onset to admission was generally short, and no patients were reported to have distant metastases. Therefore, paediatric tracheobronchial IMT may be more amenable to be completely resected at an earlier stage. Given its non-specific symptoms and rare characteristics, the paediatric tracheobronchial IMT is still more likely to be misdiagnosed. Patients were previously misdiagnosed as pneumonia in this study or asthma in other cases [4]. Therefore, we suggest that children who experience unexplained respiratory symptoms or fever and have no response to treatment should be referred to further examinations, including radiology and bronchoscopy, and tracheobronchial IMT should be aware of.

The diagnosis of paediatric tracheobronchial IMT is based on radiology and histology. When the patients are suspected of tracheobronchial IMT, CT scan is highly suggested. Tracheobronchial masses and unspecific imaging findings such as atelectasis, lung infection and pleural effusion can be observed in the CT images of paediatric tracheobronchial IMTs. CT outcomes are conducive to defining the tumour anatomical location but not for diagnostic validation, for which further pathological examination is necessary. The typical pathological characteristic of IMT is the proliferation of spindle cells, surrounded by inflammatory cells such as plasma cells, lymphocytes or eosinophils [5]. A part of paediatric tracheobronchial IMT can be diagnosed by bronchoscopic pathological outcomes, and it is easier to obtain effective pathological tissues with a rigid one [6]. Obtaining a small tissue sample for frozen sections would make pathological diagnosis difficult [7]. Meanwhile, as a low-grade malignant tumour without obvious malignant tumour characteristics, the frozen section usually shows no evidence of malignancy. Thus, sometimes the outcomes of frozen surgical sections are negative, but the surgical paraffin section is positive. The postoperative surgical paraffin section is the most reliable evidence to confirm the diagnosis. Our study reported a case as tumour-free margin according to the frozen surgical sections, but with an opposite outcome in light of the paraffin section [8]. Such an opposite outcome may lead to incomplete resection. IHC is helpful in the differential diagnosis. The ALK is one of the special indicators for IMT and >50% of IMT patients are IHC ALK positive [9]. The ALK-positive rate in our study was even higher and reached 75%. More and more ALK fusion genes were discovered in recent years, such as EML4-ALK, TPM3-ALK and PPFIBP1-ALK [10–12]. A positive ALK provides an additional option for treatment. Positive *ROS1* gene and *RET* gene expressions have also been reported [13, 14]. The ALK also has certain significance in suggesting disease metastasis and prognosis [15].

Surgical resection is the mainstay for paediatric tracheobronchial IMT. In clinical practice, lobectomy or pneumonectomy that expands the extent of resection can prevent recurrence but have relatively high morbidity and mortality [16]. Compared with lobectomy or pneumonectomy, limited surgical resection preserves more lung parenchyma [17]. Limited surgical resections include tracheal reconstruction, carinal reconstruction, sleeve lobectomy and bronchial sleeve resection. The purpose of limited resection is to achieve a negative surgical margin and preserve more lung function. Reports of limited surgical resections on paediatric tracheobronchial IMT are rare [18, 19]. In our study, all participants underwent limited surgical resection in our study, with small amounts of intraoperative blood loss without short-term postoperative complications. From our experience, the airway tumour surgical margin can be considered tumour-free when it is 5 mm above and below the tumour. Paediatric airway reconstruction is challenging because children have small chest cavities and fragile bronchial systems. As for the surgical technique, delicate handling of tissue is required to minimize scars and strictures. The use of a small-sized thoracoscope for surgery can enable a clearer surgical field of vision. The minimally invasive surgical method enables enhanced recovery. Reducing anastomotic tension is of vital significance for children. Experiments have shown that the amount of tension on tracheal suture lines tolerated by puppies was only 58% of that tolerated by adult dogs [20]. Anastomotic laceration can cause acute dyspnoea and, in severe cases, even lead to death. Dyspnoea caused by anastomotic stenosis is another problem in airway reconstruction. Before suturing, we will use thread to tie both airway ends for a test pull. Through the pull thread, the tension on both sides can be felt to adjust the angle of the anastomosis and the length of the resection. The airway diameter of children is smaller than that of adults, and small scars can cause severe stenosis. Absorbable sutures should be used for airway sutures during surgery to reduce the possibility of anastomotic stenosis. Polydioxanone plus suture which was absorbable and antibiotic was applied to the suture of the trachea and bronchus in our participants. The continuous sutures can reduce the granulation tissue formation, and the reinforcement of intermittent sutures can reduce the risk of anastomotic leakage. Thus, continuous sutures combined with intermittent sutures were used to minimize the possibility of anastomosis complications. Postoperative management of airway reconstruction is very important. Patients should be closely monitored, and complications should be detected in time. It is feasible to apply limited surgical resection in children with experienced surgeons and careful surveillance.

The adjuvant treatment of paediatric tracheobronchial IMT is mainly used for patients unwilling to receive surgery with incomplete resection or recurrence. The adjuvant treatment includes targeted therapy, radiotherapy and chemotherapy. Crizotinib has been proved effective in treating ALK-positive IMT.[21, 22] The IMT patients with *ROS1* positive also responded to crizotinib. Chemotherapy has more side effects than targeted therapy and is uncommonly used in IMT patients [23]; however, chemotherapy can be used as a treatment option for patients regardless of their gene condition. Local radiotherapy, which has few systematic side effects, can be considered when patients are unwilling to receive surgery or systemic treatment [24]. Since no official management or guidelines are available, participants in our study did not receive postoperative adjuvant therapy. According to our observation, paediatric tracheobronchial IMT receiving complete resection has an excellent prognosis even without postoperative adjuvant treatment.

**Figure 3:**
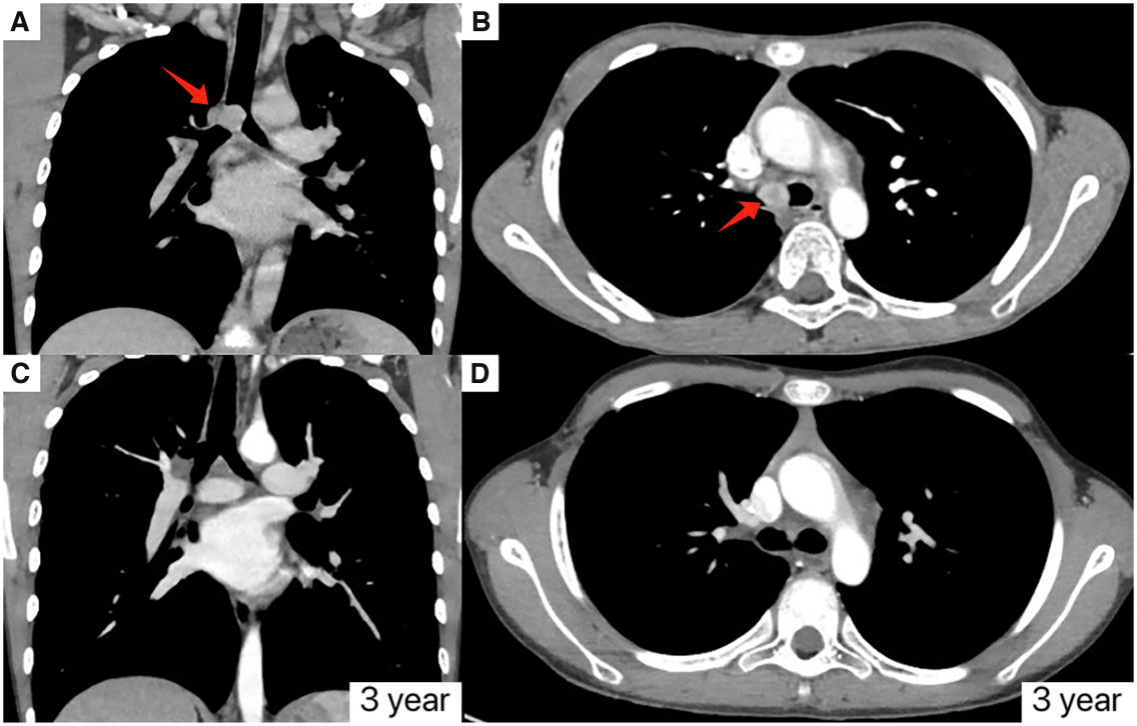
Computed tomographic image of case number 3 in this study. (**A**) Preoperative computed tomographic image (coronal view). (**B**) Preoperative computed tomographic image (axial view). (**C**) Postoperative computed tomographic image (coronal view). (**D**) Postoperative computed tomographic image (axial view).

We completely resected 8 tracheobronchial IMTs without adjuvant therapy, and none of the participants died or had a local recurrence in the follow-up. Previous studies have suggested that the 5-year survival rate of IMT undergoing complete resection is as high as 91% [25]. The prognosis of paediatric tracheobronchial IMT with complete surgical resection is excellent and has low recurrence rates. Only 1 participant with incomplete excision developed local recurrence in this study. This patient was diagnosed as recurrence with tracheal stenosis. After tumour excision combined with airway dilation, this patient recovered and did not relapse until the last follow-up. Although complete surgical resection is the best way to treat IMT, the risks of surgery should be heeded. Experienced doctors must perform paediatric tracheobronchial surgery and be closely monitored after the operation. CT scan can show well-defined endo-tracheobronchial mass. It is less time-consuming and cheap. We recommended CT scans at the first 6 months and annually after surgery. Bronchoscopy should be performed after any positive CT finding (Fig. 3).

### Limitations

There were some limitations in this study. First of all, participants did not receive postoperative spirometry to quantify the evaluation of pulmonary function. However, it is easy to understand that more lung pulmonary parenchyma preserved means better lung function and quality of life. Unfortunately, it was a retrospective study lacking a control group. Hence, a prospective study with a control group should be considered in future studies.

## CONCLUSION

In conclusion, tracheobronchial IMTs should be suspected in any paediatric patients presenting with tracheobronchial mass and unexplained respiratory symptoms. Limited surgical resection by an experienced surgeon is the preferred treatment for paediatric tracheobronchial IMT to prevent unavoidable pneumonectomy or lobectomy. After complete resection, prognosis is excellent and recurrence is rare.

## Data Availability

All data generated or used during the study appear in the submitted article.

## ABBREVIATIONS

CT: Computed tomography
IHC: Immunohistochemistry
IMT: Inflammatory myofibroblastic tumour
IQR: Interquartile range
